# The Construction of Support Vector Machine Classifier Using the Firefly Algorithm

**DOI:** 10.1155/2015/212719

**Published:** 2015-02-23

**Authors:** Chih-Feng Chao, Ming-Huwi Horng

**Affiliations:** Department of Computer Science and Information Engineering, National Pingtung University, No. 4-18, Min Sheng Road, Pingtung 90003, Taiwan

## Abstract

The setting of parameters in the support vector machines (SVMs) is very important with regard to its accuracy and efficiency. In this paper, we employ the firefly algorithm to train all parameters of the SVM simultaneously, including the penalty parameter, smoothness parameter, and Lagrangian multiplier. The proposed method is called the firefly-based SVM (firefly-SVM). This tool is not considered the feature selection, because the SVM, together with feature selection, is not suitable for the application in a multiclass classification, especially for the one-against-all multiclass SVM. In experiments, binary and multiclass classifications are explored. In the experiments on binary classification, ten of the benchmark data sets of the University of California, Irvine (UCI), machine learning repository are used; additionally the firefly-SVM is applied to the multiclass diagnosis of ultrasonic supraspinatus images. The classification performance of firefly-SVM is also compared to the original LIBSVM method associated with the grid search method and the particle swarm optimization based SVM (PSO-SVM). The experimental results advocate the use of firefly-SVM to classify pattern classifications for maximum accuracy.

## 1. Introduction

The support vector machines (SVMs) have been widely used in many applications, including the decision-making application [1], forecasting malaria transmission [2], liver fibrosis diagnosis [3], and pattern classification [4]. The SVM achieves the tradeoff between the minimum training set error and the maximization of the margin based on the Vapnik-Chervonenkis theory and structural risk minimization principle. Thus it has the best generalization ability [5–7]. Essentially, the SVM is a convex quadratic programming method with which it is possible to find the global rather than the local optima. However, the setting of the parameters for the SVM classifier plays a significant role, which includes the penalty parameter *C* and the smoothness parameter *γ* of the radial-based function. The penalty parameter *C* maintains the balance between the fitting error minimization and model complexity. The smoothness parameter *γ* of the kernel function is used to determine the nonlinear mapping from the input space to the high-dimensional feature space.

In general, the redundant features of the classifier usually significantly slow down the learning process as well as make the designed SVM classifier overfitting the training data. In general, an effective feature selection can tackle the cure-of-dimension problem as well as decrease the computation time. Practically, the grid search [8, 9] checks all possibilities of the parameters, *C* and *γ*, under exponentially growing sequences. More precisely, the search for the two parameters is limited to the intervals of the 1/2^15^ ≤ *C* ≤ 2^15^ and 1/2^5^ ≤ *γ* ≤ 2^5^. In practical application, the grid search is usually vulnerable to the local optimum. In other words, if the initial parameters *C* and *γ* are far from that global optimum, the resulting SVM classifier will not work effectively.

Recently, many bioinspired optimization algorithms such as genetic algorithm [10] and particle swarm optimization [11] have been applied to train the parameters of the SVM classifier together with a powerful features selection for classification. However, the Lagrangian multipliers *α*_*i*_ are still estimated by using a grid search similar to the LIBSVM [12]. However, those of the designed support vector machines present a challenge to be combined into a one-against-all support vector machine classifier, because each SVM always holds a different set of features in the multiclass classifications. Another algorithm, the artificial bee colony algorithm [13], was applied to only train the parameter of Lagrangian multiplier *α*_*i*_ without the parameters of *C* and *γ*.

In this paper, the firefly algorithm [14] is used to search for the optimal parameters via the simulation of the social behavior of fireflies and their phenomenon of bioluminescent communication. The proposed algorithm is called the firefly-SVM, in which all parameters including the *C*, *γ*, and the Lagrangian multiplier *α*_*i*_ are concurrently trained. In the experiments, the proposed firefly-SVM was evaluated by the classifications for binary and multiclass problems. This paper is organized as follows. The proposed firefly-SVM algorithm is introduced in Section 2. In Section 3, we present our experiments and demonstrate the results. The conclusion and final remarks are given in Section 4.

## 2. Materials and Methods

### 2.1. Support Vector Machines

The SVM has been one of the more widely used data learning tools in recent years. It is usually used to address a binary pattern classification problem. The binary SVM constructs a set of hyperplane in an infinite dimensional space, which can then be divided into two kinds of representations, such as the linear and nonlinear SVM.

First, we consider a binary classification problem; the training data set = {(*x*_1_, *y*_1_), (*x*_2_, *y*_2_), (*x*_3_, *y*_3_),…, (*x*_*l*_, *y*_*l*_)}, *y*_*i*_ ∈ {−1,1}, *x*_*i*_ ∈ *R* ^*d*^, where *x*_*i*_ is the data point and the corresponding *y*_*i*_ is its designed label. The *l* denotes the number of elements in the training data set.

The linear SVM finds the optimal separating margin by solving the following optimization task:

(1)  Minimize  12w2+C∑i=1lεi, εi≥0

(2)  Subject  to yiwTxi+b≥1−εi, i=1,2,…,l,

where *C* is a penalty value, *ε*_*i*_ are positive slack variables, **w** is a normal vector, and **b** is a scalar quantity. The minimum problem can be reduced by using the Lagrangian multiplier *α*_*i*_, which can obtain its optimum according to the Karush-Kuhn-Tucker condition. If *α*_*i*_ > 0, then the corresponding data *x*_*i*_ is called the support vector (SV), and, therefore, the linear discriminate function can be expressed with the optimal hyperplane parameters **w** and **b** in the following equation:

(3) $$\begin{array}{c} f\left(x\right)=\mathrm{sgn}\left(\overset{l}{\underset{i=1}{\sum }}\alpha_{i}y_{i}x_{i}^{T}x+b\right). \end{array}$$

Equation (1) can be transformed into (4) by its unconstrained dual form:

(4)  Maximize∑i=1lαi−12∑i,j=1lαiαjyiyjxixj,

(5)  C≥αi≥0, i=1,…,l, ∑i=1lαiyi=0.

Equation (4) can now be solved using the quadratic programming techniques and the stationary Karush-Kuhn-Tucker condition. The resulting solution **W** can be expressed as a linear combination of the training vectors and the **b** can be expressed as the average of all support vectors shown in

(6) $$\begin{array}{c} W=\overset{l}{\underset{i=1}{\sum }}\alpha_{i}y_{i}x_{i},\\ b=\frac{1}{N_{\text{SV}}}\overset{N_{\text{SV}}}{\underset{i=1}{\sum }}\left(Wx_{i}-y_{i}\right), \end{array}$$

where *N*_SV_ is the number of support vectors.

The linear SVM can be expanded into the nonlinear cases by replacing *x*_*i*_ with a mapping into the feature space *θ*(*x*_*i*_), in other words, the *x*_*i*_^*T*^ *x* can be represented as the form of *θ*(*x*_*i*_)^*T*^ *θ*(*x*_*i*_) in the feature space. Thus the nonlinear discriminate function can be expressed as follows:

(7) $$\begin{array}{c} f\left(x\right)=\mathrm{sgn}\left(\overset{l}{\underset{i=1}{\sum }}\alpha_{i}y_{i}K\left(x_{i},x\right)+b\right); \end{array}$$

where *K*(*x*_*i*_, *x*) = 〈*θ*(*x*_*i*_), *θ*(*x*)〉 and *K*(*x*_*i*_, *x*) is the kernel function. The widely used kernel function is the radial basis function (RBF), because of its accurate and reliable performance [15], which is defined as

(8) $$\begin{array}{c} K\left(x_{i},x\right)=\exp{\left(-\gamma \left\|x_{i}-x\right\|\right)}^{2}. \end{array}$$

The *γ* is the predetermined smoothness parameter that controls the width of the RBF kernel; thus, (4) is rewritten as

(9)  Maximize∑i=1lαi−12∑i,j=1lαiαjyiyjexp⁡⁡−γxi−x2, C≥αi≥0, i=1,…,l, ∑i=1lαiyi=0.

To evaluate the proposed firefly-SVM, the classification results of 10 two-class data sets of the UCI machine repository are compared to those for the LIBSVM algorithm [12] and PSO-SVM [11] in this paper.

The one-against-all (OAA) and one-against-one (OAO) strategies are widely used for the multiclass classifiers [7]. The OAA approach always compares each class with all the others put together concurrently, and, thus, it always needs the construct *k* support vector machines for a *k*-class classification problem [16]. The OAO approach [17] constructs many binary classifiers for all possible pairs of classes; therefore, it needs the construct *k*(*k* − 1)/2 support vector machines for a *k*-classification problem. A max-wins voting scheme determines its instance classification. In our past studies of the classification of the ultrasonic supraspinatus images [18], the OAA fuzzy SVM had the best capability in the classification of the ultrasonic images into different disease groups. Therefore, in this paper, the binary firefly-SVM is implemented as the basic SVM to construct the OAA fuzzy SVM for a further comparison to the original fuzzy SVMs in the classification of supraspinatus images.

### 2.2. Firefly Algorithm

The firefly algorithm is a new bioinspired computing approach for optimization in which the search mechanism is simulated by the social behavior of fireflies and the phenomenon of bioluminescent communication. There are two important issues regarding the firefly algorithm, namely, the variation of light intensity and the formulation of attractiveness. Yang [14] simplifies the attractiveness of a firefly by determining its brightness which in turn is associated with the encoded objective function. The attractiveness is proportional to the brightness. Every member *x*_*i*_ of the firefly swarm is characterized by its brightness *I*_*i*_ which can be directly measured as a corresponding fitness function.

Furthermore, there are three idealized rules: (1) regardless of their sex, any one firefly will be attracted to other fireflies; (2) attractiveness is proportional to brightness, so of any two flashing fireflies, the less bright one will move toward the brighter one; (3) brightness of a firefly is affected or determined by the landscape of the given fitness function *φ*(*x*); in other words, the brightness *I*(*x*_*i*_) of a firefly *x*_*i*_ can be defined as its *φ*(*x*_*i*_).

More precisely, the attractiveness between fireflies *x*_*i*_ and *x*_*j*_ is defined as any monotonically decreasing function as shown in (10), for their distance *r*_*ij*_:

(10) $$\begin{array}{c} r_{i,j}=\left\|x_{i}-x_{j}\right\|=\sqrt{\overset{c}{\underset{k=1}{\sum }}{\left(x_{i,k}-x_{j,k}\right)}^{2}},\\ \beta =\beta_{0}e^{-{\gamma r}_{i,j}}, \end{array}$$

where *β*_0_ is the sum of initial assigned brightness of these two fireflies. *γ* is the light absorption coefficient and *k* is the index of the dimension of the candidate solutions (i.e., fireflies).

The movement of a firefly *x*_*i*_, which is attracted to another more attractive firefly *x*_*j*_, is determined by the following equations:

(11) $$\begin{array}{c} x_{i,k}=\left(1-\beta \right)x_{i,k}+{\beta x}_{j,k}+u_{i,k},\\ u_{i,k}=\left(\text{rand}1-\frac{1}{2}\right). \end{array}$$

If there is no firefly brighter than a particular firefly, *x*_*i*^max⁡^_, it will move randomly according to the following equation:

(12) $$\begin{array}{c} x_{i^{\max},k}=x_{i^{\max},k}+u_{i^{\max},k},\\ u_{i^{\max},k}=\left(\text{rand}2-\frac{1}{2}\right), \end{array}$$

where rand1 and rand2 are random numbers obtained from the uniform distribution *U*(0, 1).

### 2.3. Training the Nonlinear SVM Using the Firefly Algorithm

The training of the nonlinear SVM is essentially a constrained optimization problem. The constrained optimization usually first decides the objective function (i.e., fitness function in the firefly algorithm) and the range of each parameter. The designed fitness function of the firefly-SVM is expressed in the following equation:

(13) MAX Lαi,C,γ =∑i=1Nαi−12∑i,j=1Nαiαjyiyjexp⁡⁡−γxi−x2.

The constraints of the solution string are

1. 0 ≤ *α*_*i*_ ≤ *C*,  *i* = 1,…, *N*, and ∑_*i*=1_^*N*^ *α*_*i*_ *y*_*i*_ = 0,
−15 ≤ log⁡_2_ *C* ≤ 15,
−5 ≤ log⁡_2_ *γ* ≤ 5.

**Figure 1:**
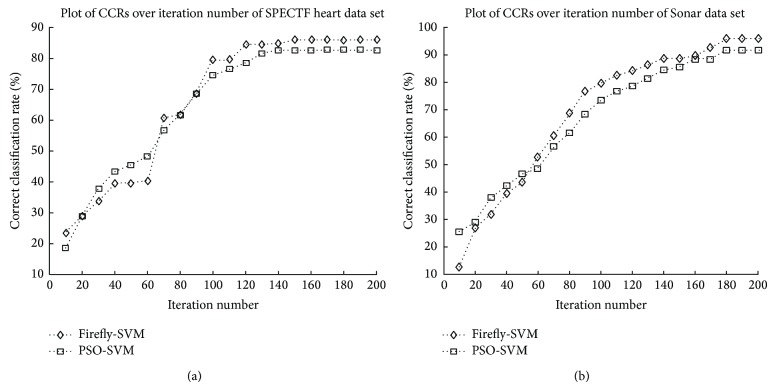
The plots of correct classification rate versus iteration numbers of SPECTF heart and Sonar data sets.

From these discussions, it is clear that the firefly algorithm starts with a set of firefly population (candidate solutions) in the feature space. The string representation *S*_*i*_ of each firefly (solution) *f*_*i*_ is an important factor for the subsequent steps of the algorithm; the solution string *S*_*i*_ is simulated as the multidimensional vector comprising optimization parameters, including the penalty parameter, smoothness parameter, and Lagrangian multipliers shown in Figure 1. It is evident that each firefly during the course of the search modifies its path according to its brightness. Furthermore, the best firefly performs the random walk to exchange it with a brighter solution. The firefly populations of m initial solutions are generated with *n* + 2 dimensions denoted by **D**:

(14) $$\begin{array}{c} D=\left[S_{1},S_{2},S_{3},\ldots ,S_{m}\right],\\ S_{i}=\left(\alpha_{1}^{i},\alpha_{2}^{i},\alpha_{3}^{i},\ldots ,\alpha_{n}^{i},\log_{2}C^{i},\log_{2}\gamma^{i}\right), \end{array}$$

where 0 ≤ *α*_*k*_^*i*^ ≤ *C*, ∑_*k*=1_^*N*^ *α*_*k*_^*i*^ *y*_*i*_ = 0, −15 ≤ log⁡_2_ *C* ^*i*^ ≤ 15, and −5 ≤ log⁡_2_ *γ* ^*i*^ ≤ 5. The *α*_*k*_^*i*^ is the multiplier of *k*th training data in the *i*th candidate solution; log⁡_2_ *C* ^*i*^ and log⁡_2_ *γ* ^*i*^ are the penalty parameter and smooth parameter of the SVM, respectively, constructed by the solution string *S*_*i*_.

The details of the proposed algorithm are thus described as follows.

**Step 1 (set up the parameters of proposed system).**

This step assigns the parameters including the number of fireflies (*m*), the maximum iteration number (*l*), and the light absorption coefficient (*γ*).  The solution string of *m* fireflies is randomly generated; it must satisfy the constraint of (13). Let *t* be iteration number and initiated to 0. The initial brightness *β*_0_^*i*^  of each firefly *f*_*i*_ is assigned by its resulting fitness.

**Step 2 (update all candidate solutions).**

The mechanism for updating a candidate solution is stochastic; that is, the solution *S*_*i*_ randomly selects the corresponding solution from this population** D**. If the fitness (*S*_*i*_) is less than fitness (*S*_*j*_), the firefly *f*_*i*_ will move toward the firefly *f*_*j*_; as a result, the corresponding string *S*_*i*_ is modified according to the following equation:

(15) Si,k=1−βSi,k+βSj,k+wki,hhhhhhhhfor  all  dimension  k,

where

1. ${\;r}_{i,j}=\left\|S_{i}-S_{j}\right\|=\sqrt{\sum_{k=1}^{n+2}{\left(S_{i,k}-S_{j,k}\right)}^{2}}$,
2. where *s*_*i*,*k*_ is the *k*th dimension of the solution string *S*_*i*_,
3. *β* = *β*_*d*_ *e* ^−*γr*_*i*,*j*_^,
4. where *β*_*d*_ is the sum of the fitness values of the two solutions of these two fireflies, and *γ* is the light absorption coefficient,
5. w=(w1i,w2i,…,wn+2i) a random walk ranged with −1 < *w*_*k*_^*i*^ < 1.

If the new solution does not satisfy the solution string constraints, then the new solution will be discarded, or else the original one will be replaced. All candidate solutions will sequentially be updated according to the previous procedure, and then the best one will be calculated for the later process. The best solution will be recorded by *u*_BestCu_.

**Step 3 (update the best solution).**

If the *d*th firefly is the *u*_BestCu_, then this firefly will demonstrate a random walk to get the new candidate solution but it still needs to satisfy the solution constraints. If the new one has better fitness than the original one, then the *u*_BestCu_ will be replaced by the new candidate solution; otherwise the candidate solution will be discarded:

(16) $$\begin{array}{c} S_{d,k}=S_{d,k}+w_{k}^{d},\;\forall k, \end{array}$$

where *w* = (*w*_1_^*d*^, *w*_2_^*d*^, …, *w*_*n*+2_^*d*^) a random walk ranged with −1 < *w*_*k*_^*d*^ < 1.

**Step 4 (iterative execution and resulted vector output).**

Add *t* by 1. If *t* reaches the maximum iteration number, then the algorithm terminates and outputs the *u*_BestCu_ as the resulting motion vector; otherwise go to Step 2.

## 3. Results and Discussion

### 3.1. Binary Classification of the UIC Data Set

The designed platform used to develop the firefly-SVM training algorithm was a personal computer with the Intel Pentium IV 3.0 GHz CPU, 2 GB RAM, using the Window XP operating system and the Visual C++ 6.0 together with an OPENCV library environment. The used parameters are the size of initial fireflies assigned to be 20 and the maximum iteration number to be 200. In order to obtain classification results without partiality, the ten binary class data sets extracted from the UIC database [19] are applied to the experiments listed in Table 1. In practice, the features with the different numeric ranges always dominate those of small numeric ranges; thus each feature is linearly scaled into the range [−1,1] for all data samples.

In all experiments on binary classifications, the fivefold cross validation method is used. In practice, all data samples are divided into fivesubsets with an equal number of samples from each different class. One of thefive subsets is selected as the test set, and the other 4 subsets are put together to form a training set. More precisely, every sample appears in a test set exactly once and appears in the training set four times. In order to verify the effectiveness of the proposed firefly-SVM, the correct classification ratio (CCR) is used for all the data sets. The CCR is defined as follows:

(17) $$\begin{array}{c} \text{CCR}=\frac{\text{number}\;\text{of}\;\text{correct}\;\text{decisions}}{\text{Total}\;\text{number}\;\text{of}\;\text{data}\;\text{samples}}\times 100. \end{array}$$

The Matthews correlation coefficient (MCC) [20] is a powerful measure of the quality of the binary classifier; it takes into account true and false positives and is generally regarded as a balanced measure that can be used even if the classes are of different sizes. MCC is defined in the following equation:

(18) MCC =TP×TN−FP×FNTP+FP×TP+FN×TN+FP×TN+FN.

In this equation, TP is the number of true positives, TN is the number of true negatives, FP is the number of false positives, and FN is the number of false negatives. MCC returns a value between −1 and +1, and it is in essence a correlation coefficient between the observed and the predicted binary classification. Therefore, a Matthews correlation coefficient of +1 indicates a perfect prediction, 0 indicates no better than random prediction, and −1 presents total disagreement between the prediction and the observation.

Table 2 shows the CCRs of 10 data sets of UCI data repository using firefly-SVM, the PSO-SVM without a feature selection, and the original LIBSVM with grid search method. The PSO-SVM trains the parameters of *C* and *γ* and then classifies all samples of each data set into two different classes, using LIBSVM, while the LIBSVM uses the grid search to find the appropriate parameters of *C* and *γ*. As shown in Table 2, the CCR of firefly-SVM is superior to both PSO-SVM and LIBSVM for all data sets. In particular, for data sets with Australian credit approval and SPECTF heart, the proposed firefly-SVM performance exceeds a 4% correct classification rate when compared to the other two methods.

Table 3 shows the Matthews coefficients of 10 data sets using the three different classifiers. The results of Table 3 reveal that the firefly-SVM is almost greater than the other two methods, while the low MCCs for the data sets of SPECTF heart, the Pima-Indians-diabetes, and the liver disorder reveal that firefly-SVM still has possible room for improvement with further study.

In order to ensure the convergences of firefly-SVM and PSO-SVM algorithms, the plots of CCRs versus the iteration number of executions of SPECTF heart and the Sonar data sets are shown in Figure 1. The resulting CCRs using the corresponding parameters in intervals of 10 iterations running the firefly-SVM and PSO-SVM are recorded. From the results of Figure 1, we find that the program converges when it runs less than 200 iterations. The total training time for firefly-SVM is about 6.68 seconds, and the training time for PSO-SVM is 5.36 seconds.

Furthermore, we attempt to discuss whether or not the classifier, together with the feature selection mechanism, can improve the classification by using the SVM classifier. In general, the irrelevant or redundant features usually lead to overfitting and even to poor accuracy of the classification. In the firefly-SVM algorithm we use the mutual information as the search criterion to find the powerful features in each data set. In practical applications, the features of all samples of each data set are evaluated using a mutual information criterion, and then the features with higher mutual information are selected as input features for the firefly-SVM algorithm. The detailed algorithms of the mutual information feature selection can be referred to as [18, 21]. However, the PSO-SVM of Table 4 integrates the feature selection mechanism, which is used for searching the parameters of *C* and *γ*, into the objective function in the training stage using the PSO searching algorithm [22]. Table 4 shows the CCR results of the firefly-SVM and the PSO-SVM classifiers with the feature selection mechanism. Table 4 shows that the CCRs of 10 data sets using the PSO-SVM and firefly-SVM deliver better results than the other results in Table 2.

### 3.2. Multiclass Classification of the Ultrasonic Supraspinatus Images

In general, the injury of supraspinatus always causes shoulder pain, especially rotator cuff diseases. The ultrasonography is the most frequently used image modality to assess the damage from supraspinatus. According to Neer's diagnosis standards [23], the impingement syndrome diseases of supraspinatus can be divided into three disease groups, namely, tendon inflammation, calcific tendonitis, and supraspinatus tear. Similar to the experiments in a past study [21], the ultrasonic image database used was recorded from 2004 to 2008, and the ages of the patient ranged from 15 to 65 years. The 120 images in this database were captured using an HDI Ultramark 5000 ultrasound system (ATL Ultrasound, CA, USA) with the ATL linear array probe from the National Cheng King University Hospital. These images are divided into four disease groups, that is, normal, tendon inflammation, calcific tendonitis, and tear. In our past referenced studies [18], five multiclass support vector machine algorithms were employed to classify these images. These five methods were original OAA SVM (OAA-SVM), OAA fuzzy SVM (OAA-FSVM), OAA decision-tree based SVM (OAA-DTB), one-against-one voting based SVM (OAO-VB), and one-against-one directed acyclic group SVM (OAO-DAG). The experimental results of that previous work showed that the CCR of the OAA-FSVM is the best method for the classification of supraspinatus images. The original OAA-FSVM is composed of many binary support vector machines; each support vector machine is trained by LIBSVM, together with a grid search. Similar procedures for feature extraction from supraspinatus, feature selection, and feature normalization were described in [18]. Five powerful texture features, sum average, sum variance, mean convergence, contrast, and difference variance, were used as the features for classification. Furthermore, many measure indices, such as sensitivity, specificity, and *F*-score, are discussed in [24].

In the current experiments, we replaced this LIBSVM based support vector machine with firefly-SVM for comparison.  Table 5 shows the performance indices of OAA-FSVM with different trained support vector machines based on the 5-fold cross validation. Referring to Table 5 we find that the false negative using the firefly-SVM is only 2.5%, which is better than the one for LIBSVM. This means that the OAA-FSVM using the firefly-SVM as constructed basis has a lower risk for the patient in diagnosis. At the same time, the 92.5% accuracy of firefly-SVM based OAA-FSVM is superior to the original OAA-FSVM trained by LIBSVM.  Table 6 shows the performance indices for the use of firefly-SVM based OAA-FSVM trained by LIBSVM and the original OAA-FSVM trained by LIBSVM. This table shows that the firefly-SVM based OAA-FSVM performs better.

## 4. Conclusion

In this paper, we explore the uses of the firefly-SVM for binary and multiclass classification. Based on the results of the current experiments on the binary classification of 10 data sets of the UCI database and the multiclass classification of ultrasonic supraspinatus images, the following conclusions can be emphasized.

1. The firefly-SVM attempts to simultaneously train three kinds of parameters: penalty parameter, smoothness parameter, and Lagrangian multiplier. Experimental results demonstrate that firefly-SVM is capable of dealing with the applications of pattern classification.
2. The firefly-SVM training algorithm has better performance than the other two methods in the experiments of binary classification, so it is promising to apply firefly-SVM to other practical problems.
3. The firefly-SVM may converge with the most optimal solution within a limited time when it associates with the feature selection because of its complexity. Additionally, the firefly-SVM without a feature selection easily and extensively integrates with the multiclass OAA support vector machine, such as the OAA-FSVM method. The experimental results of the classification of ultrasonic supraspinatus images reveal that the use of the firefly-SVM as the basic machine to construct the multiclass support vector machine can effectively improve the classification performances in the multiclass classification of ultrasonic supraspinatus images.

## Figures and Tables

**Table 1:** The used data set extracted from the UCI machine learning data repository.

Data set collected from UCI the machine learning repository		
Data set	Number of instances	Number of features
SPECTF heart	267	44
Breast Cancer Wisconsin (diagnosis)	569	32
Statlog (heart)	270	13
German credit data	1000	20
Sonar	208	60
Pima-Indians-diabetes	768	8
Australian credit approval	690	14
Live disorders	345	7
Ionosphere	351	34
Breast Cancer Wisconsin (original)	699	10

**Table 2:** The CCR of three different algorithms without feature selection (mean ± SD).

Data set	Firefly-SVM	PSO-SVM	LIBSVM
SPECTF heart	84.27 ± 0.95	80.19 ± 2.44	80.19 ± 2.73
Breast Cancer Wisconsin (diagnosis)	98.44 ± 0.39	97.54 ± 0.90	97.54 ± 1.54
Statlog (heart)	86.75 ± 2.19	86.75 ± 2.0	85.25 ± 2.99
German credit data	77.60 ± 0.89	75.33 ± 2.61	76.34 ± 1.24
Sonar	93.46 ± 3.31	92.69 ± 4.18	89.23 ± 4.63
Pima-Indians-diabetes	77.87 ± 1.33	76.34 ± 2.59	72.25 ± 3.22
Australian credit approval	88.63 ± 1.40	84.34 ± 3.77	83.76 ± 3.36
Live disorders	75.36 ± 2.99	73.22 ± 3.28	69.86 ± 2.67
Ionosphere	96.87 ± 1.91	95.21 ± 2.84	94.35 ± 2.25
Breast Cancer Wisconsin (original)	97.60 ± 0.37	96.62 ± 0.96	95.52 ± 0.92

**Table 3:** The Matthews correlation coefficient for Table 2.

Data set	Firefly-SVM	PSO-SVM	LIBSVM
SPECTF heart	0.5104	0.4453	0.4592
Breast Cancer Wisconsin (diagnosis)	0.9663	0.9475	0.9598
Statlog (heart)	0.9962	0.9242	0.9019
German credit data	0.4981	0.3977	0.4721
Sonar	0.8488	0.8358	0.7865
Pima-Indians-diabetes	0.5580	0.5258	0.4206
Australian credit approval	0.7707	0.6417	0.6734
Liver disorders	0.4967	0.4529	0.3869
Ionosphere	0.9374	0.9032	0.8863
Breast Cancer Wisconsin (original)	0.9462	0.9242	0.9019

**Table 4:** Classification results of the firefly-SVM and PSO-SVM with feature selection.

Data sets	* *Number of original features	Firefly-SVM		PSO-SVM	
		CCR (mean ± SD)	Number of features	CCR (mean ± SD)	Average number of features
SPECTF heart	44	85.89 ± 1.43	18	82.34 ± 2.174	22.6
Breast Cancer Wisconsin (diagnosis)	32	98.44 ± 0.39	14	98.44 ± 0.90	13.4
Statlog (heart)	13	86.75 ± 2.19	8	87.51 ± 2.21	8.6
German credit data	20	79.34 ± 1.14	12	75.33 ± 2.23	14.3
Sonar	60	95.23 ± 3.41	20	91.31 ± 2.42	32.7
Pima-Indians-diabetes	8	78.83 ± 1.62	5	77.87 ± 1.49	5.4
Australian credit approval	14	89.43 ± 1.65	8	84.34 ± 4.77	8.6
Liver disorders	7	75.36 ± 2.29	4	74.76 ± 2.12	5.2
Ionosphere	34	97.98 ± 3.15	12	95.21 ± 2.14	17.6
Breast Cancer Wisconsin (original)	10	98.67 ± 0.53	6	98.67 ± 1.39	6.7

**Table 5:** Performance evaluation for each disease group.

Method used	Sensitivity (%)	Specificity (%)	False negative rate (%)	Accuracy (%)
Method 1: firefly-SVM based OAA-FSVM			2.5	92.50 ± 1.37
(1) Normal	93.10	90.00		
(2) Inflammation tendon	93.10	96.42		
(3) Calcific tendon	96.67	93.10		
(4) Supraspinatus tear	100.00	100.00		

Method 2: original OAA-FSVM trained by LIBSVM			3.33	89.10 ± 2.49
(1) Normal	83.33	95.56		
(2) Inflammation tendon	86.67	95.56		
(3) Calcific tendon	90.00	96.67		
(4) Supraspinatus tear	100.00	100.00		

**Table 6:** Performance indices for the firefly-SVM based and LIBSVM based OAA-FSVM.

Measures	Firefly-SVM based OAA-FSVM	LIBSVM based OAA-FSVM
Accuracy (%)	92.5	89.1
Sensitivity (%)	96.6	90.0
Specificity (%)	87.1	86.0
Youden's index^*^ (%)	83.7	76.0
*F*-score	95.9	92.6

^*^Youden's index = Sensitivity + Specificity – 1.
